# Allogenic Platelet-Rich Plasma for the Treatment of Adhesive Capsulitis

**DOI:** 10.7759/cureus.47491

**Published:** 2023-10-22

**Authors:** Ashim Gupta, Adarsh Aratikatla, Scott M Martin

**Affiliations:** 1 Regenerative Medicine, Regenerative Orthopaedics, Noida, IND; 2 Regenerative Medicine, Future Biologics, Lawrenceville, USA; 3 Regenerative Medicine, BioIntegrate, Lawrenceville, USA; 4 Orthopaedics, South Texas Orthopaedic Research Institute, Laredo, USA; 5 Medicine, The Royal College of Surgeons in Ireland, Dublin, IRL; 6 Medical Aesthetics, Elite Medical Aesthetics, Las Vegas, USA

**Keywords:** synovitis, frozen shoulder, adhesive capsulitis, regenerative medicine, allogenic prp, prp, platelet-rich plasma

## Abstract

Adhesive capsulitis (AC) is a common shoulder disorder leading to pain and restricted range of motion (ROM) and affects the patient’s activities of daily living (ADL) and overall quality of life (QoL). Conservative therapies are prioritized, resorting to surgical intervention only when necessary. Unfortunately, these modalities have limitations and do not address the underlying pathological cause of AC. The use of autologous biologics, such as platelet-rich plasma (PRP), has evolved and shown promise for managing musculoskeletal (MSK) injuries, including AC. However, subpar functional outcomes have led clinicians to question the long-term efficacy of autologous PRP. To circumvent this, the possibility of utilizing a standardized and well-characterized allogenic PRP for AC has been explored. In this manuscript, we qualitatively present in vitro, pre-clinical, clinical, and ongoing studies investigating the varied applications of allogenic PRP for the management of AC. The results demonstrated that allogenic PRP acts in a pleiotropic manner and decreases pro-inflammatory cytokines only in the inflammatory condition. In addition, the administration of allogenic PRP is safe and potentially efficacious, in terms of reducing pain and improving range of motion, shoulder strength, and function, in non-surgical management of AC. Nonetheless, more pre-clinical studies and adequately powered, multicenter, prospective, non-randomized, and randomized controlled trials with longer follow-up are warranted to further establish the safety and efficacy of allogenic PRP and justify its routine clinical use.

## Introduction and background

Musculoskeletal (MSK) injuries impact billions of people throughout the world annually and can permanently affect their quality of life (QoL) [1]. Shoulder pain is the third leading MSK condition, and its incidence rises with age, with a lifetime prevalence of roughly 70%. Adhesive capsulitis (AC), also called frozen shoulder, is one of the most common shoulder disorders with a frequency of 2%-5% in the general population and more common in those aged 40-59 years [2,3]. Adhesive capsulitis is associated with contracture of the joint capsule and the rotator interval, inclusive of the superior glenohumeral ligament and the coracohumeral ligament [4,5]. Frozen shoulder classically transitions through stages of “freezing” that may last up to 24 months [6]. While initially thought to be self-limiting, current studies suggest that a significant number of patients suffer from persistent, severe discomfort and loss of function [6]. As AC leads to not only chronic pain but also restricted range of motion (ROM) of the glenohumeral joint in external rotation followed by abduction in the plane of scapula and flexion, the patient’s activities of daily living (ADL) and overall QoL are adversely impacted [7,8]. This leads to tremendous healthcare-related and socioeconomic impacts on society as a whole [2,9]. Although the etiology of AC is still unknown, several factors, including preceding synovitis, previous trauma, increasing age, female gender, and diabetes, may portend worse outcomes [7]. Current literature suggests that chronic inflammation within the capsular sub-synovial layer may progress to capsular fibrosis, contracture, and adherence to the anatomic neck of the humerus [4,10-13]. Multiple inflammatory cytokines have also been implicated in the development of this inflammatory contracture, including transforming growth factor-beta (TGF-β), tumor necrosis factor-alpha (TNF-α), and interleukins [4]. In addition, there is no consensus on the best options for the management of AC [14]. Current treatment options to manage AC include the use of pharmacological agents such as non-steroidal anti-inflammatory drugs, intra-articular steroid injections, non-pharmacological options such as physical therapy, manipulation under anesthesia, hydrodilatation, and ultimately surgical capsular release when all conservative options have been exhausted [15]. These traditional treatment modalities have flaws and side effects and normally provide short-term symptomatic relief as opposed to addressing the underlying pathologies associated with AC [16,17].

Over the last decade, there has been an increased interest in the use of autologous allografts, including platelet-rich plasma (PRP) for MSK regenerative medicine applications [18]. Numerous studies, including case reports and studies, randomized controlled trials (RCT), systematic reviews, and meta-analyses have demonstrated the safety and efficacy of PRP for MSK disorders [18-22]. Conversely, there are studies demonstrating subpar outcomes [18,23]. These are often attributed to lack of standardization, mischaracterization of PRP formulations, and patient-related variables such as age, mental and physical stress levels, smoking status, alcohol consumption, comorbidities such as diabetes, and concomitant pain medication that confounded results and threw the efficacy of PRP for AC into question [18,23]. To overcome the shortcomings presented by autologous PRP, the potential of using well-characterized allogenic PRP formulation with standardized preparation protocol has been investigated in patients with AC. The primary objective of this study is to report the in vitro, pre-clinical, and clinical outcomes of allogenic PRP for the management of AC. The secondary objective is to document the ongoing clinical trials registered on various clinical trial protocol repositories associated with allogenic PRP for the treatment of AC.

## Review

Search criteria

**Figure 1:**
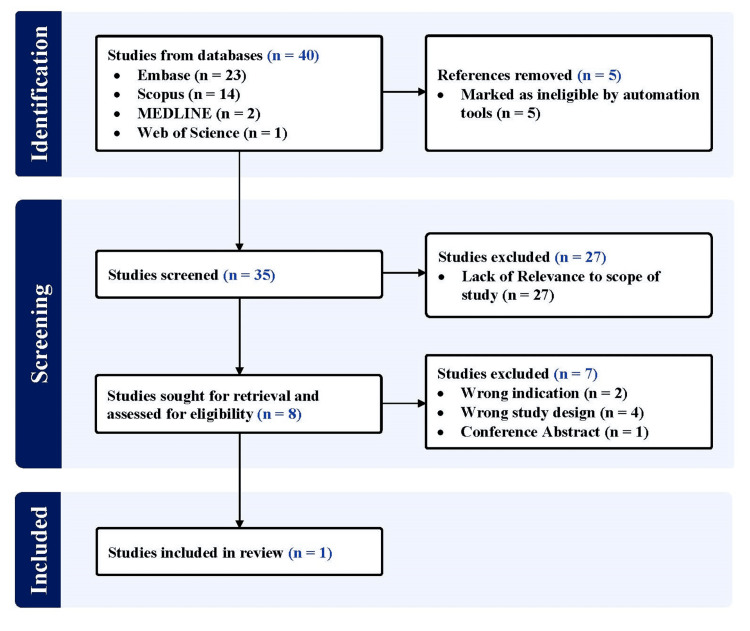
PRISMA flow diagram outlining the record identification and selection process PRISMA: Preferred Reporting Items for Systematic Reviews and Meta-Analyses

A systematic search on Embase, Scopus, MEDLINE (PubMed), and Web of Science was conducted, aiming to retrieve relevant articles published in English until September 22, 2023. Adherence to the Preferred Reporting Items for Systematic Reviews and Meta-Analyses (PRISMA) statement and guidelines was maintained throughout the study, using the following designated search terms: (“allogenic” OR “allogeneic” OR “heterologous” OR “allograft”) AND (“platelet rich plasma” OR “platelet concentrate” OR “platelet rich growth factors” OR “platelet rich fibrin”) AND (“adhesive capsulitis” OR “frozen shoulder” OR “shoulder contracture” OR “periarthritis”). All studies utilizing allogenic PRP for AC were included. Studies not using allogenic PRP or not focusing on the management of AC were excluded. All duplicates were removed by the automation tools. Figure 1 illustrates the systematic search performed.

Additionally, we searched ClinicalTrials.gov, Chinese Clinical Trial Register (ChiCTR), and Clinical Trials Registry - India (CTRI) using the aforementioned search terms to identify registered trials on the use of allogenic PRP for the management of AC.

Results

In Vitro Studies

Lee et al. [24] assessed the effect of allogenic PRP on synoviocytes with or without interleukin-1β (IL-1β)-induced inflammation. PRP was formulated using a plateletpheresis system with a leukoreduction set. Platelet count was concentrated to 4000 x 10^3^ platelets/µL (PRP4000) and then diluted to 1000 x 10^3^ platelets/µL (PRP1000) and 200 x 10^3^ platelets/µL (PRP200). Calcium gluconate (10%) was used as an activator. Synoviocytes were isolated, harvested, and cultured for 2-5 passages from patients undergoing arthroscopic rotator cuff repair. The synoviocytes were allowed to attach for 24 hours and were cultured in Dulbecco’s Modified Eagle Medium supplemented with 1% fetal bovine serum (FBS) for 24 hours. The cells were then treated with 1 ng/mL of IL-1β, 1 µM of dexamethasone, 10% vol/vol platelet-poor plasma (PPP), and 10% vol/vol PRPs in FBS-free medium for 24 hours and harvested for further assays. Non-treated synoviocytes were used as controls. Gene expression analysis via real-time reverse transcriptase polymerase chain reaction (RT-PCR) for pro-inflammatory cytokines, degradative enzymes and their inhibitors, and anti-inflammatory cytokines was performed.

The characteristics for PPP were 3.71 ± 1.25 x 10^3^ platelets/µL, no red blood cells (RBCs)/µL, and 0.01 ± 0.01 x 10^3^ white blood cells (WBCs)/µL; PRP200 were 205.33 ± 12.66 x 10^3^ platelets/µL, 0.04 ± 0.01 x 10^6^ RBCs/µL, and 0.01 ± 0.01 x 10^3^ WBCs/µL; PRP1000 were 909.00 ± 92.77 x 10^3^ platelets/µL, 0.17 ± 0.02 x 10^6^ RBCs/µL, and 0.01 ± 0.01 x 10^3^ WBCs/µL; and PRP4000 were 3440.67 ± 1000.24 x 10^3^ platelets/µL, 0.51 ± 0.08 x 10^6^ RBCs/µL, and 0.02 ± 0.03 x 10^3^ WBCs/µL.

For pro-inflammatory cytokines, without IL-1β treatment, PRP1000 and PRP4000 significantly induced the expression of pro-inflammatory cytokines IL-6, COX-2, and mPGES-1 and inhibited the expression of tumor necrosis factor-alpha (TNF-α). With IL-1β treatment, the expression of IL-1β, TNF-α, IL-6, COX-2, and mPGES-1 were significantly upregulated. Treatment with dexamethasone led to a significant decrease in the expression of IL-1β, IL-6, COX-2, and mPGES-1 and no effect on the expression of TNF-α. Treatment with PPP and PRP led to significant downregulation in the expression of IL-1β, TNF-α, IL-6, and COX-2. For degradative enzymes and their inhibitors, without IL-1β treatment, PPP, and PRP200, PRP1000 and PRP4000 induced the expression of MMP-3, TIMP-3, and ADAMTS-4. PPP decreased, while PRP increased the expression of MMP-1. PRP decreased the expression of MMP-9. PPP and PRP significantly decreased the expression of ADAMTS-5. No differences in the expression of TIMP-1 were reported. With IL-1β treatment, the expression of MMP-1, MMP-3, MMP-9, MMP-13, ADAMTS-4, and ADAMTS-5 were significantly upregulated. Treatment with dexamethasone resulted in a significant decrease in the expression of MMP-1, MMP-3, MMP-9, MMP-13, and ADAMTS-4 and an increase in the expression of ADAMTS-5. PRP1000 led to downregulation in the expression of ADAMTS-5. PPP and PRP significantly downregulated the expression of MMP-1, MMP-3, MMP-13, and ADAMTS-4. IL-1β treatment also upregulated the expression of TIMP-1 and TIMP-3. Dexamethasone, PPP, and PRP200 treatments led to a significant decrease in the expression of TIMP-3. For anti-inflammatory cytokines, without IL-1β treatment, PRP decreased the expression of IL-4, and PRP4000 increased the expression of VIP and IL-1Ra. With IL-1β treatment, the expression of IL-4 was significantly reduced, while the expression of IL-10, VIP, and IL-1Ra were increased. Treatment with PPP or PRP led to significant upregulation in the expression of VIP. In addition, PPP and PRP did not affect the expression of IL-1Ra, but the expression was significantly higher compared to corticosteroids.

Pre-clinical Studies

Thus far, there are no published pre-clinical studies involving the use of allogenic PRP for the treatment of AC.

Clinical Studies

Lee et al. [24] evaluated the safety and efficacy of allogenic PRP in patients with AC in comparison with propensity score-matched control patients treated with corticosteroids. The inclusion criteria included patients ≥18 years of age, unilateral shoulder pain of <1 year, and diagnosis of AC (painful shoulder with restricted both active and passive ROM ≥ 25% in at least two directions compared to contralateral shoulder or normal values). Fifteen patients who met the inclusion criteria were enrolled in the allogenic PRP group. All injections were administered to the patient in a seated position with the arm rotated internally in front of the abdomen under ultrasonography. Injection of either 4 mL of allogenic PRP1000 (characterization described above) or propensity-score matched controls, 1 mL of triamcinolone acetonide (40 mg/mL) in 3 mL saline, was administered into the glenohumeral joint space. Patient-reported outcome measures (PROMs) were recorded at baseline and at one week, one month, three months, and six months post-injection. Clinical outcome measures included pain, ROM (active forward flexion, abduction, external rotation with the arm at the side, and internal rotation), muscle strength (supraspinatus, infraspinatus, and subscapularis muscles), function, and overall satisfaction. No adverse events were reported with allogenic PRP throughout the duration of the study. The allogenic PRP group showed a significant reduction in pain till six months, whereas the steroid group showed faster pain reduction till three months and an increase thereafter until six months. Both groups showed significant improvement in ROM at all follow-up time points compared to baseline except for external rotation at six months; however, this improvement did not meet the values for the contralateral shoulder at any time point. The allogenic PRP group showed an increase in strength for supraspinatus and subscapularis muscles till six months, while the corticosteroid group showed an increase in strength for infraspinatus and subscapularis muscles till three months, followed by a decrease until six months. In addition, the allogenic PRP group showed improvement in all functional scores till six months, while the corticosteroid group showed more gradual improvement till three months, followed by deterioration until six months. For overall satisfaction, the Single Assessment Numeric Evaluation (SANE) score showed a statistically significant increase in the PRP group at both three and six months, whereas, in the corticosteroid group, there was a statistically significant increase till three months, followed by a decrease until six months. There was no statistical difference in improvement between the two groups at six months follow-up for either function or overall satisfaction.

Ongoing Studies

As of September 22, 2023, there are no ongoing clinical trials registered on ClinicalTrials.gov, Chinese Clinical Trial Register (ChiCTR), or Clinical Trials Registry - India (CTRI) to study the effects of allogenic PRP for the treatment of AC.

Discussion

The present study evaluated the therapeutic potential of allogenic PRP for the management of AC. In vitro, pre-clinical, and clinical studies focusing on the effect of allogenic PRP on AC were included. No pre-clinical studies are identified using the pre-defined search criteria, and no ongoing clinical trials are registered on any clinical trial protocol registries.

AC is known to be associated with the expression of IL-1α, IL-1β, IL-6, IL-8, TNF-α, COX-1, COX-2, etc., as these cytokines may play a vital role in the inflammation of synovium [25]. Studies have demonstrated that PRP inhibits the expression of pro-inflammatory cytokines including IL-1β, TNF-α, and IL-6 under stimulation of lipopolysaccharides or TNF-α [26,27]. In contrast, some studies have reported an increase in the expression of pro-inflammatory cytokines including IL-1β and IL-6, which can be attributed to the catabolic effects of leukocytes in the PRP formulation [28,29]. The pure allogenic PRP utilized by Lee et al. [24] lacked these catabolic enzymes and resulted in the downregulation of pro-inflammatory cytokines including IL-1β, TNF-α, IL-6, COX-2, and mPGES-1, specifically under inflammatory conditions. In addition, the expression of TNF-α in synoviocytes was also downregulated post-treatment with allogenic PRP. Furthermore, in AC, there is an imbalance in the matrix synthesis and degradation, which can lead to the failure of matrix remodeling [25,30]. Matrix metalloproteinases (MMPs) and their inhibitors play an essential role in matrix remodeling, and a lack of homeostasis in the MMP-to-tissue inhibitor metalloproteinase (TIMP) ratio can result in joint capsule fibrosis. Overexpression of MMPs is harmful as it can lead to the degradation of the extracellular matrix (ECM) along with mediating the downstream signaling of inflammation and apoptosis [31]. Synoviocytes, in addition, are a major contributor to the composition and function of synovial fluid, and thus, MMPs released from it can impact surrounding tissues leading to a malicious cycle in the shoulder [32]. Lee et al. [24] demonstrated that treatment with pure allogenic PRP led to the downregulation of the expression of MMP-1, MMP-3, MMP-13, ADAMTS-4, and ADAMTS-5; however, no significant changes were reported in the expression of their inhibitor, TIMP-1. This suggests that PRP can modulate the homeostasis in matrix remodeling by downregulating the IL-1β-induced overexpressed levels of MMPs along with decreasing the harmful effects on adjacent tissues. These results provide important insights into the use of allogenic PRP during inflammatory conditions only, instead of in non-inflammatory or health conditions.

Lee et al. [24] further reported that the administration of allogenic PRP is safe. The allogenic PRP significantly ameliorated pain and improved ROM, shoulder strength, and function similar to corticosteroid in patients with AC at six months follow-up. However, these outcome measures improved promptly at 1-3 months post-injection of corticosteroid, but the effect did not last till six months. On the other hand, the allogenic PRP group showed consistent improvement and reached a peak at six months of follow-up. The results from this study are in accordance with other studies that reported the short-term efficacy of corticosteroids [33,34] and better improvement in the autologous PRP group compared to corticosteroids [35,36].

This study has several limitations including a small sample size, lack of randomization, and short follow-up. Despite constraints, the aforementioned clinical study demonstrated that the administration of allogenic PRP is safe and potentially efficacious in patients with AC.

## Conclusions

In spite of our thorough search of multiple electronic databases, based on our search criteria and pre-defined inclusion/exclusion criteria, only one study with both in vitro and clinical components fit the scope of our manuscript.

The in vitro component of the identified study demonstrated the pleiotropic effect of allogenic PRP and reduced pro-inflammatory cytokines in the presence of inflammation. The clinical component showed that the administration of allogenic PRP is safe and potentially efficacious in terms of reducing pain and improving ROM and function similar to corticosteroid till three months but was superior to corticosteroid till six months. Given the dearth of relevant literature, pre-clinical studies to better understand the mechanism of action and adequately powered, multicenter, non-randomized, and randomized controlled trials with longer follow-up are warranted to further evaluate the efficacy of allogenic PRP in patients with AC.
